# Increased prevalence of *pfdhfr* and *pfdhps* mutations associated with sulfadoxine–pyrimethamine resistance in *Plasmodium falciparum* isolates from Jazan Region, Southwestern Saudi Arabia: important implications for malaria treatment policy

**DOI:** 10.1186/s12936-020-03524-x

**Published:** 2020-12-02

**Authors:** Aymen M. Madkhali, Hesham M. Al-Mekhlafi, Wahib M. Atroosh, Ahmad Hassn Ghzwani, Khalid Ammash Zain, Ahmed A. Abdulhaq, Khalid Y. Ghailan, Alkhansa A. Anwar, Zaki M. Eisa

**Affiliations:** 1 Department of Medical Laboratory Technology, Faculty of Applied Medical Sciences, Jazan University, Jazan, Kingdom of Saudi Arabia; 2 Medical Research Centre, Jazan University, Jazan, Kingdom of Saudi Arabia; 3 Department of Parasitology, Faculty of Medicine, University of Malaya, 50603 Kuala Lumpur, Malaysia; 4 Department of Parasitology, Faculty of Medicine and Health Sciences, Sana’a University, Sana’a, Yemen; 5 Department of Microbiology and Parasitology, Faculty of Medicine and Health Sciences, University of Aden, Aden, Yemen; 6 Faculty of Public Health and Tropical Medicine, Jazan University, Jazan, Kingdom of Saudi Arabia; 7 Saudi Centre for Disease Prevention and Control, Ministry of Health, Jazan, Kingdom of Saudi Arabia

**Keywords:** Malaria, *Plasmodium falciparum*, Artemisinin-based combination therapy, Drug resistance, Infectious diseases, Saudi Arabia

## Abstract

**Background:**

Despite significant progress in eliminating malaria from the Kingdom of Saudi Arabia, the disease is still endemic in the southwestern region of the country. Artesunate plus sulfadoxine–pyrimethamine (AS + SP) has been used in Saudi Arabia since 2007 as a first-line treatment for uncomplicated *Plasmodium falciparum* malaria. This study aimed to investigate the prevalence of mutations associated with resistance to artemisinin and sulfadoxine–pyrimethamine (SP) resistance in *P. falciparum* parasites circulating in Jazan region, southwestern Saudi Arabia.

**Methods:**

A total of 151 *P. falciparum* isolates were collected between April 2018 and March 2019 from 12 of the governorates in Jazan region. Genomic DNA was extracted from dried blood spots and amplified using nested PCR. Polymorphisms in the propeller domain of the *P. falciparum* k13 (*pfkelch13*) gene and point mutations in the *P. falciparum* dihydrofolate reductase (*pfdhfr*) and dihydropteroate synthase (*pfdhps*) genes were identified by sequencing.

**Results:**

No mutations in the *pfkelch13* propeller domain were found in any of the 151 isolates. However, point mutations in the *pfdhfr* and *pfdhps* genes were detected in 90.7% (137/151) of the isolates. The *pfdhfr* double mutations N51**I** + S108**N** (i.e. AC**I**C**N**I haplotype) and triple mutations N51**I** + C59**R** + S108**N** (i.e. AC**IRN**I haplotype) were detected in 47% and 37.8% of the isolates, respectively. Moreover, the *pfdhps* single mutation at codon A437**G** and double mutations A437**G** + K540**E** (i.e. S**GE**AAI haplotype) were observed in 4.6% and 51.7% of the isolates, respectively. Interestingly, 23.8%, 25.1 and 12.6% of the isolates had quintuple, quadruple and triple mutated combined *pfdhfr*–*pfdhps* genotypes, respectively. Furthermore, significant associations were found between the prevalence of mutant haplotypes and the age, gender and nationality of the patients (*P* < 0.05).

**Conclusion:**

This study revealed a high prevalence of point mutations in the *pfdhfr* and *pfdhps* genes of *P. falciparum* isolates from Jazan region, with quintuple and quadruple mutant *pfdhfr*–*pfdhps* genotypes reported for the first time in Saudi Arabia and the Arabian Peninsula. Despite the absence of the *pfkelch13* mutation in the isolates examined, the *pfdhfr* and *pfdhps* mutations undermine the efficacy of SP partner drug, thereby threatening the main falciparum malaria treatment policy in Saudi Arabia, i.e. the use of AS + SP. Therefore, the continuous molecular and in-vivo monitoring of ACT efficacy in Jazan region is highly recommended.

## Background

Malaria is one of the most common tropical infectious diseases worldwide. It is particularly prevalent in the tropics and subtropics. The global burden and economic cost of the disease are still immense: approximately 220 million cases of malaria occur annually worldwide, and in 2018 alone there were nearly half a million malaria-related deaths [1, 2]. Human malaria is caused by four *Plasmodium* species: *Plasmodium falciparum*, *Plasmodium vivax*, *Plasmodium malariae* and *Plasmodium ovale*. Of these species, *P. falciparum* is considered the most virulent and prevalent, accounting for 99.7%, 62.8% and 69% of the malaria cases reported by the World Health Organization (WHO) Regional Offices for Africa, South-East Asia and the Eastern Mediterranean, respectively [1].

One of the critical challenges to malaria elimination is the emergence and spread of anti-malarial drug resistance. In response to widespread chloroquine resistance, artemisinin-based combination therapy (ACT) has been adopted by the WHO as the first-line anti-malarial treatment for uncomplicated *P. falciparum* malaria [3]. The artemisinin-based combinations used consist of an artemisinin derivative [artesunate (AS) or artemether] co-administered with a longer-acting partner drug [e.g. sulfadoxine–pyrimethamine (SP) or lumefantrine or mefloquine] [4, 5]. In some regions, ACT is supplemented with a single, low-dose primaquine for clearance of *P. falciparum* gametocytes [6]. ACT has remained highly efficacious for the last 2 decades, but is now facing a major threat due to the emergence and spread of *P. falciparum* strains resistant to both artemisinin and its partner drugs, a phenomenon first observed in the western region of Cambodia in 2009 [7, 8]. Mutations at the propeller domain of the Kelch 13 protein encoded by the *P. falciparum* k13 (*pfkelch13*) gene have been associated with delayed parasite clearance due to resistance to artemisinin [9, 10]. In addition, allelic mutations in *P. falciparum* dihydrofolate reductase (*pfdhfr*) and dihydropteroate synthetase (*pfdhps*) genes have also been associated with resistance to pyrimethamine and sulfadoxine, respectively [11, 12]. It has also been demonstrated that an accumulation of mutant *pfdhps* and *pfdhfr* alleles confers clinical resistance to SP. Of these, the quintuple mutant genotype that includes *pfdhfr* (N51**I** + C59**R** + S108**N**) and *pfdhps* (A437**G** + A540**E**) has been found to be significantly associated with in-vivo resistance to SP [13, 14].

In Saudi Arabia, the malaria control programme, which was established in 1956, has achieved tremendous progress in reducing the incidence of malaria cases and interrupting local malaria transmission [15, 16]. Major progress was seen between 2000 and 2010, when the number of indigenous cases reduced significantly from 511 in 2000 to only 29 in 2010, with the number of imported cases remaining at around 1500 per annum [17]. Thus, the country has been included in the E-2020 WHO initiative with the aim of achieving a target of zero indigenous cases by 2020 [1]. However, the numbers have increased, and 5382 cases were reported in 2016, including 272 indigenous cases, mostly in Jazan and Aseer regions [1]. The first cases of resistance to chloroquine and SP monotherapies were documented in Jazan in 1997 and 2007, respectively [18, 19]. Thus, ACT was adopted in 2007 for the treatment of uncomplicated *P. falciparum* malaria, with AS + SP or artemether–lumefantrine (AL) as first- and second-line treatments, respectively [20, 21]. Overall, there is a scarcity of information on the molecular markers of anti-malarial drug resistance in Saudi Arabia. While limited studies have investigated selected point mutations for SP resistance [19, 22], just one recent study has assessed the presence of polymorphism in the *pfkelch13* propeller domain, but only in 13 *P. falciparum* isolates collected from Taif region, western Saudi Arabia [23].

Thus, this study aimed to investigate the prevalence and distribution of the mutations present in the *pfkelch13* propeller domain, and in the *pfdhfr* and *pfdhps* genes of the *P. falciparum* parasites circulating in Jazan region. Regular assessment of the molecular markers for anti-malarial drug resistance is crucial to the success of malaria control programmes and can help health authorities and policy-makers to predict the effectiveness of and resistance to anti-malarial drugs and thus respond in a timely manner to the emergence of resistance.

## Methods

### Study area and patients

**Fig. 1:**
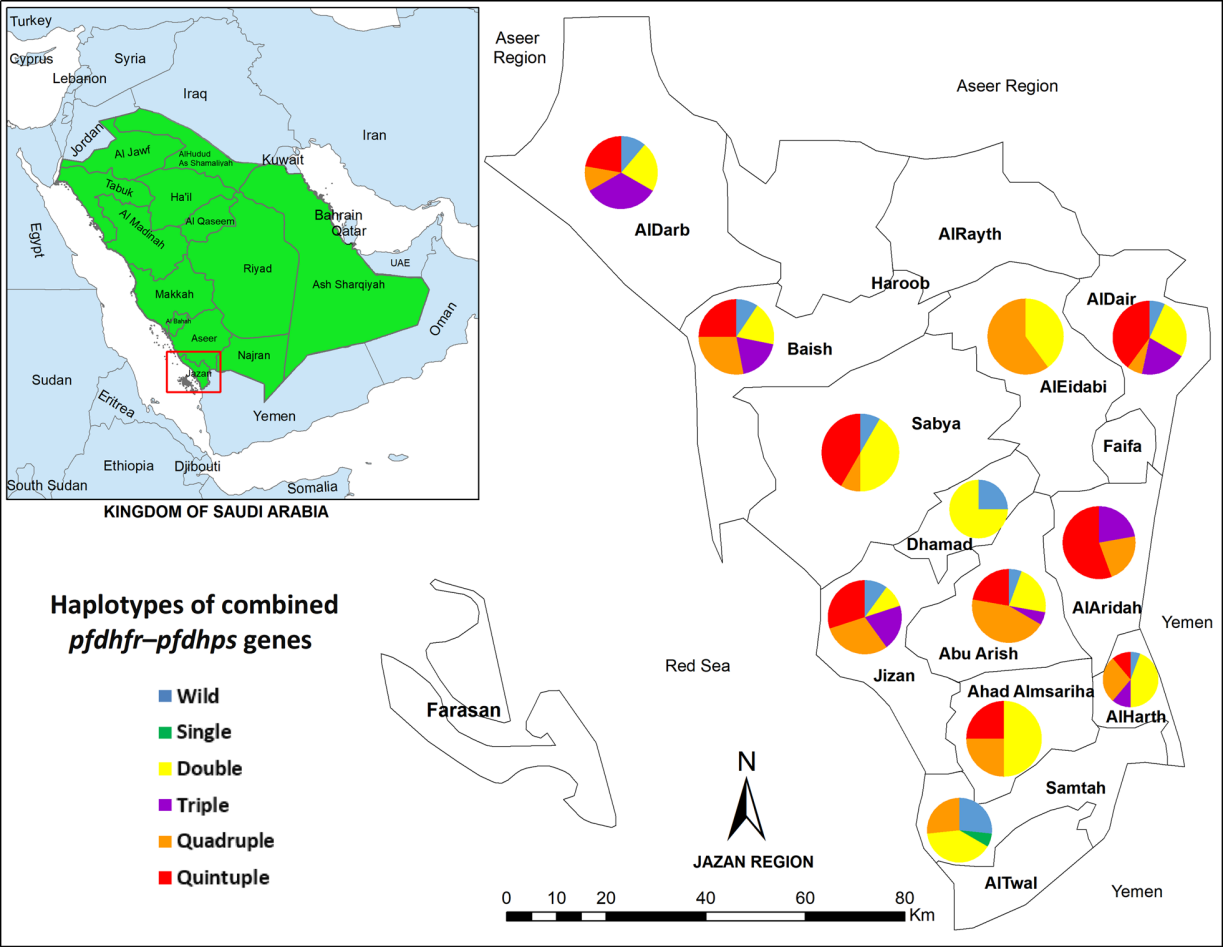
A geographic map showing study area (Jazan region, southwestern Saudi Arabia) and the distribution of the combined *pfdhfr*–*pfdhps* mutant haplotypes across the 12 governorates involved in this study. The map was created using the Esri ArcGIS 10.7 software

A hospital-based cross-sectional survey targeting individuals with fever, who were thus suspected to have malaria, was carried out between April 2018 and March 2019 in Jazan region, Saudi Arabia. Jazan region (16° 17′ North, 42° 43′ East), which is the second smallest region (after Al Baha region) in Saudi Arabia, is located in the far southwestern part of the country, 1140 km from Riyadh, the capital. It is bordered by Yemen to the south and to the west 300 km of coastline is bordered by the Red Sea. Covering a total area of 11,671 km^2^, it is the most populated region in the country, with an estimated population of 1.4 million [24]. The region is divided into 17 governorates including Jizan, the capital city (Fig. 1). All governorates were included in this study.

Generally, Saudi Arabia has a desert climate. However, the country’s climate differs from one region to another due to the variety in the topography. Jazan region can be divided into three distinct areas: (1) the highlands at an elevation of more than 2000 m above sea level and rainfall of more than 300 mm/year; (2) the foothills at an elevation of 400–600 m above sea level and rainfall of less than 300 mm/year; and (3) the coastal plains at an altitude of less than 400 m above sea level and rainfall of less than 100 mm/year. The climate varies from hot with high humidity in the coastal areas to cold and less humid in the highlands. The temperature ranges from 40 °C in June–July to 31 °C in January–February.

There are a few streams in the region as well as 12 dams for agricultural irrigation [24]. Malaria is still endemic in the region, and while transmission occurs in some areas year-round, there is a transmission peak between December and March. *Anopheles arabiensis* and *Anopheles sergentii* are the two principal malaria vectors present in Jazan [25].

The target population was febrile individuals attending public hospitals in all governorates. Blood samples were collected from patients as part of their diagnosis and healthcare. The minimum sample size required for this study was estimated according to the formula provided by [26]. At a 5% significance level and confidence level of 95%, the minimum sample size required was estimated as 384. Generally, the prevalence of malaria in the region is very low [25]. However, a prevalence of 50% was assumed because of uncertainty about the prevalence among febrile patients in the region. Overall, 530 patients participated in the study and 151 *P. falciparum* isolates were included in the molecular analysis.

### Data collection

At the diagnostic laboratories of the involved hospitals, a sample of approximately 3 mL of venous blood was collected from each patient into a labelled EDTA tube. Directly, a drop of the blood was tested for malaria using a rapid diagnostic kit (AMP Rapid Test—Malaria p.f./pan, AMEDA Labordiagnostik GmbH, Austria). Simultaneously, thick and thin blood smears were prepared on clean and properly labelled glass slides. In addition, blood spots were also obtained on labelled 3MM Whatman® filter papers (Whatman International Ltd, Maidstone, England). The blood spots were air dried in a clean place and then stored properly in individual zipped plastic bags at 4–6 °C until use.

### Microscopic examination

The thin and thick blood films of patients who tested positive for malaria by the rapid diagnostic test were stained with diluted Giemsa and then examined microscopically for confirmation and parasitaemia estimation. The parasite species was identified and parasitaemia was estimated by counting the asexual stages against 300 leukocytes and then multiplying by 25, under the assumption of a mean total white blood cell count of 7500 cells per μL of blood [27]. The level of parasitaemia (parasite density) was categorized into three levels: low (< 1000 parasites/μL of blood), moderate (1000–9999 parasites/μL of blood), and high (≥ 10,000 parasites/μL of blood). For quality control, 25% of the slides were randomly selected and re-examined by another specialist.

### Molecular analysis

Two to three discs (6 mm diameter) of the dried blood spots were cut using a sterile puncher and each was placed into a 1.5-mL microcentrifuge tube for the purpose of DNA extraction, which was performed using a Qiagen blood and tissue kit (QIAGEN, DNeasy® Blood & Tissue Kit, Cat. no. 69506, Germany) following the manufacturer’s instructions and the procedures described in a previous study [27]. Before any molecular analysis for resistant markers was undertaken, all *P. falciparum* positive samples were confirmed by using a PCR assay that targeted the small subunit ribosomal RNA genes, according to the Singh protocol [28].

The genomic DNA samples that had been purified from the *P. falciparum* isolates were amplified by conventional or nested PCR to detect selected domains of the parasite genes (*pfkelch13*, *pfdhfr* and *pfdhps*) that are known to be associated with SP-based ACT drug resistance. The mutations were identified by direct sequencing. Details of the primer sequences used for the *pfkelch13*, *pfdhfr* and *pfdhps* genes have been described previously [27].

### Detection of* pfkelch13* mutations

A single-run PCR was performed using specific primers designed to obtain an amplicon corresponding to nucleotides 1094–2127 (codons 364–709) [27]. A 50-µL reaction consist of 20 µL of ExPrime Tag Premix (GENET BIO, Korea), 200 nM of each primer and 2 µL of parasite genomic DNA template. The amplification reaction was initiated by 5 min of denaturation at 94 °C followed by 40 cycles (30 s of denaturation at 94 °C, 90 s of annealing at 60 °C and a 90 s extension at 72 °C) and a final extension step of annealing at 72 °C for 10 min. The yielded amplicons were visualized using 1% agarose gel stained with Sybr® safe DNA gel stain (Invitrogen, USA) under UV. The gel at a specific band (1062 bp) was cut, purified and sequenced and then aligned to *kelch13* gene of the *P. falciparum* 3D7 strain (PF3D7_1343700, Gene ID: 814205) located at chromosome 13 (NC: 004331.3) using BioEdit Sequence Alignment Editor Software (version 7.1.9) and Molecular Evolutionary Genetics Analysis (Mega) software (version 7.0.26). All positions were screened and assessed for the presence of potential mutations.

### Detection of *pfdhfr* and *pfdhps* mutations

The *pfdhfr* gene was examined for potential point mutations at six codons (A16V, N51I, C50R, C59R, S108N and I164L) through nested PCR amplification according to protocols described previously [27]. The primary amplification reaction was done using an Amp1 and Amp2 oligonucleotide pair primer while the secondary reaction was performed using an SP1 and SP2 primers [27]. The gel band of 700 bp was cut, purified and sequenced. Similarly, the *pfdhps* gene was also examined at six codons (S436A/F, A437G, K540E, A581G, A613T/S, and I640F) using nested PCR according to the protocols described previously [27, 28]. The PCR amplification, targeting a larger amplicon of 1005 bp, was done using PS1 + O2 and PSA + O2 oligo primer pairs for the primary and secondary reactions, respectively.

The primary PCR was carried out in 25 µL containing 10 µL of ExPrime Tag Premix (GENET BIO, Korea), 200 nM of each primer and 1 µL of genomic DNA. 2 µL of the primary PCR product was used as a template for secondary reaction in a 50 µL reaction: 20 µL of the Premix and 200 nM of each primer. For *pfdhfr*, both amplification reactions were initiated by denaturation at 94 °C for 5 min followed by 30 cycles (25 cycles for the secondary reaction) of denaturation, annealing and extension (94 °C/30 s, 49 °C/1 min and 72 °C/1 min, respectively) followed by a final extension at 72 °C for 5 min. The PCR cycling conditions were the same for *pfdhps* except that denaturation was performed at 94 °C for 1 min and annealing at 56 °C for 2 min. All PCR products were then processed for visualization using 1.5% agarose gels. The yielded amplicons were subjected to purification and sequencing. Then, the obtained sequences were aligned to the gene sequences of the *P. falciparum* 3D7; *pfdhfr* gene (PF3D7_0417200, Gene ID: 9221804) which is located at Chromosome 4; NC_004318.2 and *pfdhps* (PF3D7_0810800, Gene ID: 2655294) located at Chromosome 8 (NC_004329.3) using the BioEdit Sequence Alignment Editor Software (version 7.1.9) and Molecular Evolutionary Genetics Analysis (Mega) software (version 7.0.26).

### Data analysis

Data analysis was performed using IBM SPSS version 20 (IBM Corp., NY, USA). Distribution of the categorical variables including the point mutations of the codons and other factors such as gender and nationality were presented as frequencies and proportions. In addition, the associations of allelic point mutations with the independent variables including age, gender, nationality and parasitaemia level were examined using the Chi-square test and Fisher’s exact test, where applicable. A *P* value of < 0.05 was considered as the level of statistical significance for all tests.

## Results

One hundred and fifty-one blood spots samples were examined by PCR and confirmed positive for *P. falciparum*. Of these, 128 (84.8%) were from male and 23 (15.2%) were from female patients, with a mean ± SD age of 30.0 ± 11.0 years. Majority of the patients (64; 42.4%) aged 18–30 years followed by those aged 31–40 years (41; 27.2%) while 19 patients (12.6%) aged below 18 years. The patients were from 12 out of the 17 governorates of Jazan region, with about one fifth (21.2%) of the patients coming from Baish governorate followed by Abu Arish and Alharth governorates with 11.9% each. About one third (34.4%; 52/151) of the patients were Saudi while 65.6% (99/151) were non-Saudi, with patients from Yemen representing the majority of non-Saudis (65.7%; 65/99). With regards to parasitaemia, 53.6% of the patients had a low level of parasitaemia while 46.4% had moderate-to-high parasitaemia.

### Molecular markers of drug resistance

#### *Plasmodium falciparum kelch 13* propeller

Sequences for *pfkelch13* propeller domain were successfully obtained from all of the 151 PCR-positive malaria samples and analysed for the presence of point mutations. It was found that none of the study samples carried any mutations at the 40 codons previously found to be associated with *P. falciparum* parasite resistance to artemisinin [29, 30].

#### *Plasmodium falciparum* dihydrofolate reductase and dihydropteroate synthase genes

A total of 151 isolates were genotyped for *pfdhfr* and *pfdhps* (Table 1). Of these, 128 (84.8%) showed at least a single point mutation in the *pfdhfr* gene. Mutations at codons N51**I** and S108**N** were predominant, with 84.8% each followed by C59**R** (37.8%; 57/151) (Table 2). However, no mutations were detected at other codons (A16**V**, C50**R** and I164**L**).

**Table 1:** Frequency distribution of *pfdhfr* and *pfdhps* point mutations for *P. falciparum* isolates from Jazan, Saudi Arabia (n = 151)

Marker^a^	Number	%
*Pfdhfr*		
A16**V**		
Wild	151	100
Mutated	0	0
C50**R**		
Wild	151	100
Mutated	0	0
N51**I**		
Wild	23	15.2
Mutated	128	84.8
C59**R**		
Wild	94	62.3
Mutated	57	37.7
S108**N**		
Wild	23	15.2
Mutated	128	84.8
I164**L**		
Wild	151	100
Mutated	0	0
*Pfdhps*		
S436**A**/**F**		
Wild	151	100
Mutated	0	0
A437**G**		
Wild	66	43.7
Mutated	85	56.3
K540**E**		
Wild	73	48.3
Mutated	78	51.7
A581**G**		
Wild	151	100
Mutated	0	0
A613**T**/**S**		
Wild	151	100
Mutated	0	0
I640**F**		
Wild	151	100
Mutated	0	0

^a^Mutant alleles are bold and underlined

**Table 2:** Frequency of haplotypes of *pfdhfr, pfdhps*, and combined *pfdhfr*–*pfdhps* genes in *P. falciparum* isolates from Jazan, Saudi Arabia (n = 151)

Gene/haplotype^a^	Type of mutations	N (%)
*Pfdhfr*		
ACNCSI	Wild	23 (15.2)
AC**I**C**N**I	Double	71 (47.0)
AC**IRN**I	Triple	57 (37.8)
*Pfdhps*		
SAKAAI	Wild	66 (43.7)
S**G**KAAI	Single	7 (4.6)
S**GE**AAI	Double	78 (51.7)
*Pfdhfr*–*pfdhps*		
ACNCSI–SAKAAI	Wild	14 (9.3)
ACNCSI–S**G**KAAI	Single	1 (0.7)
ACNCSI–S**GE**AAI	Double	8 (5.3)
AC**I**C**N**I–SAKAAI	Double	35 (23.2)
AC**I**C**N**I–S**G**KAAI	Triple	2 (1.3)
AC**IRN**I–SAKAAI	Triple	17 (11.3)
AC**IRN**I–S**G**KAAI	Quadruple	4 (2.6)
AC**I**C**N**I–S**GE**AAI	Quadruple	34 (22.5)
AC**IRN**I–S**GE**AAI	Quintuple	36 (23.8)

^a^Mutant alleles are bold and underlined

With regard to *pfdhps*, 56.3% (85/151) of the isolates showed at least a single point mutation in the *pfdhps* gene. The two major mutations, A437**G** and K540**E**, were observed in 85 (56.3%) and 78 (51.7%) isolates, respectively while all other codons (S436**A**/**F**, A581**G**, A613**T**/**S** and I640**F**) were of the wild type.

Table 2 shows the frequency of *pfdhfr* and *pfdhps* allelic haplotypes. Nine allelic haplotypes were identified, with the *pfdhfr* AC**I**C**N**I haplotype predominant (47.0%) followed by the AC**IRN**I haplotype (37.8%) and the wild-type ACNCSI haplotype (15.2%). On the other hand, the S**GE**AAI *pfdhps* haplotype was predominant (51.7%) over the S**G**KAAI haplotype (4.6%). Interestingly, genotyping of the 151 *P. falciparum* isolates for the combined *pfdhfr*–*pfdhps* genes revealed that 61.5% of the isolates had triple or more allelic mutations, with 23.8%, 25.1 and 12.6% of the isolates having quintuple, quadruple and triple mutated genotypes, respectively. Half (18/36) of the isolates with quintuple mutated genotypes were found in Saudi patients followed by 11 (30.6%) in Yemeni patients. On the other hand, nearly half (18/38; 47.4%) of the isolates with quadruple mutant genotypes were found in isolates from Yemeni patients followed by 11 (28.9%) from isolates from Saudi patients.

Table 3 shows that frequency of the *pfdhfr* triple mutated haplotype (AC**IRN**I) was significantly higher in isolates collected from patients aged ≥ 30 years compared to those from patients aged below 30 years (48.7% vs 26.7%; *P* = 0.005). Similarly, the frequency of the *pfdhps* double mutant haplotype (S**GE**AAI) was significantly higher in isolates collected from female compared those from male participants (73.9% vs 47.7%; *P* = 0.020). With regard to nationality, the frequency of the *pfdhfr* double mutant haplotype (AC**I**C**N**I) was significantly higher in isolates collected from non-Saudi compared to those from Saudi patients (55.6% vs 30.8%; *P* = 0.004), whereas the frequency of the *pfdhfr* triple mutated haplotype (AC**IRN**I) was higher in isolates collected from Saudi than non-Saudi patients (50.0% vs 31.3%; *P* = 0.024). Interestingly, the frequency of the *pfdhfr*–*pfdhps* quintuple haplotype (AC**IRN**I–S**GE**AAI) was significantly higher in isolates collected from patients aged ≥ 30 years compared to those from patients aged below 30 years (34.2% vs 13.3%; *P* = 0.003), and in isolates collected from Saudi than non-Saudi patients (34.6% vs 18.2%; *P* = 0.024). Similarly, the frequency of the AC**I**C**N**I–S**GE**AAI quadruple haplotype was significantly higher in isolates collected from female compared those from male participants (39.1% vs 19.5%; *P* = 0.038). On the other hand, no significant association was found between parasitaemia and the distribution of *pfdhfr* and *pfdhps* haplotypes (*P* > 0.05).

**Table 3:** Frequency of *pfdhfr* and *pfdhps* mutant alleles and related haplotypes for *P. falciparum* isolates from Jazan, Saudi Arabia according to demographic factors and parasitaemia (n = 151)

Marker^a^	Total	Age group			Gender			Nationality			Parasitaemia		
		< 30	≥ 30	*P*	Females	Males	*P*	Saudi	Non-Saudi	*P*	Low	Moderate-to-high	*P*
*Pfdhfr*													
ACNCSI	23 (15.2)	16 (21.3)	7 (9.2)	0.038	1 (4.3)	22 (17.2)	0.203^†^	10 (19.2)	13 (13.1)	0.322	10 (12.3)	13 (18.6)	0.288
AC**I**C**N**I	71 (47.0)	39 (52.0)	32 (42.1)	0.223	13 (56.5)	58 (45.3)	0.321	16 (30.8)	55 (55.6)	0.004	41 (50.6)	30 (42.9)	0.341
AC**IRN**I	57 (37.8)	20 (26.7)	37 (48.7)	0.005	9 (39.1)	48 (37.5)	0.882	26 (50.0)	31 (31.3)	0.024	30 (37.0)	27 (38.6)	0.846
*Pfdhps*													
SAKAAI	66 (43.7)	35 (46.7)	31 (40.8)	0.467	6 (26.1)	60 (46.9)	0.064	19 (36.5)	47 (47.5)	0.198	37 (45.7)	29 (41.4)	0.601
S**G**KAAI	7 (4.6)	3 (4.0)	4 (5.3)	0.988^b^	0 (0.0)	7 (5.5)	0.596^b^	5 (9.6)	2 (2.0)	0.048^b^	3 (3.7)	4 (5.7)	0.705^b^
S**GE**AAI	78 (51.7)	37 (49.3)	41 (53.9)	0.571	17 (73.9)	61 (47.7)	0.020	28 (53.8)	50 (50.5)	0.696	41 (50.6)	37 (52.9)	0.784
*Pfdhfr*–*pfdhps*													
ACNCSI–SAKAAI	14 (9.3)	8 (10.7)	6 (7.9)	0.557	0 (0)	14 (10.9)	0.129^b^	7 (13.5)	7 (7.1)	0.241^b^	6 (7.4)	8 (11.4)	0.396
ACNCSI–S**G**KAAI	1 (0.7)	1 (1.3)	0 (0)	0.497^b^	0 (0)	1 (0.8)	0.848^b^	0 (0)	1 (1.0)	0.656^b^	0 (0)	1 (1.4)	0.464^b^
ACNCSI–S**GE**AAI	8 (5.3)	7 (9.3)	1 (1.3)	0.034^b^	1 (4.3)	7 (5.5)	0.649^b^	3 (5.8)	5 (5.1)	0.562^b^	4 (4.9)	4 (5.7)	0.557^b^
AC**I**C**N**I–SAKAAI	35 (23.2)	18 (24.0)	17 (22.4)	0.812	4 (17.4)	31 (24.2)	0.475	8 (15.4)	27 (27.3)	0.101	22 (27.2)	13 (18.6)	0.212
AC**I**C**N**I–S**G**KAAI	2 (1.3)	1 (1.3)	1 (1.3)	0.748^b^	0 (0)	2 (1.6)	0.718^b^	1 (1.9)	1 (1.0)	0.572^b^	0 (0)	2 (2.9)	0.213^b^
AC**IRN**I–SAKAAI	17 (11.3)	9 (12.0)	8 (10.5)	0.775	2 (8.7)	15 (11.7)	0.502^b^	4 (7.7)	13 (13.1)	0.315	9 (11.1)	8 (11.4)	0.951
AC**IRN**I–S**G**KAAI	4 (2.6)	1 (1.3)	3 (3.9)	0.620^b^	0 (0)	4 (3.1)	0.513^b^	4 (7.7)	0 (0)	0.103^b^	3 (3.7)	1 (1.4)	0.624^b^
AC**I**C**N**I–S**GE**AAI	34 (22.5)	20 (26.7)	14 (18.4)	0.225	9 (39.1)	25 (19.5)	0.038	7 (13.5)	27 (27.3)	0.054	19 (23.5)	15 (21.4)	0.766
AC**IRN**I–S**GE**AAI	36 (23.8)	10 (13.3)	26 (34.2)	0.003	7 (30.4)	29 (22.7)	0.420	18 (34.6)	18 (18.2)	0.024	18 (22.2)	18 (25.7)	0.616

All values are number (%)

Parasitaemia levels: low (< 1000 parasites/μL of blood); moderate-to-high (≥ 1000 parasites/μL of blood)

Significant association (*P* < 0.05)

^a^Mutant alleles are bold and underlined

^b^The difference was examined using Fisher’s exact test (otherwise, Chi-square test was used)

Additional file 1: Table S1 shows significant associations between all detected *pfdhfr* point mutations (N51**I**, C59**R** and S108**N**) and age groups (*P* < 0.05), between C59**R** *pfdhfr*-mutant allele and nationality (*P* = 0.024), and between K540**E** *pfdhps* mutant allele and gender (*P* = 0.020). Moreover, Fig. 1 and Additional file 1: Table S2 show the frequency and distribution of the *pfdhfr*, *pfdhps*, and combined *pfdhfr*–*pfdhps* mutant haplotypes across the governorates involved in this study. The highest percentage of quintuple mutant haplotypes was found in AlAridah governorate (55.6%) followed by Sabya (41.7%) and AlDair (40.0%). On the other hand, the frequency of quadruple mutant haplotypes was highest in AlEidabi governorate (60.0%) followed by Abu Arish (44.4%) and Jizan (30.0%).

## Discussion

In this study, the prevalence and distribution of polymorphisms associated with *P. falciparum* resistance to AS + SP, the first-line ACT in Saudi Arabia, were investigated. The investigation focused on the point mutations within the *pfkelch13*, *pfdhfr* and *pfdhps* genes. Despite intensive efforts to eliminate malaria from Saudi Arabia, active foci for transmission are still reported in Aseer and Jazan regions. Both regions have diverse ecological and natural environments that favour vector breeding sites and local malaria transmission [16, 31]. Moreover, both regions share borders with Yemen, a country with a high malaria transmission rate [32, 33]. Between 2015 and 2017, over 32% of all imported malaria cases reported in all regions of Saudi Arabia were of Yemeni origin [34].

This study demonstrated that all isolates analysed for the potential polymorphism in *pfkelch13* propeller gene were found of wild type showing no mutations throughout the amplified sequences. This finding is consistent with the only previous study on potential mutations in *pfkelch13* in isolates, which was conducted in the Taif region of Saudi Arabia [23]. Similar findings have also been reported in other countries either in the Arabian Peninsula or in the East Mediterranean Region including Yemen, Sudan and Somalia [27, 35, 36]. On the other hand, few non-synonymous *pfkelch13* mutations were identified in *P. falciparum* isolates from other neighbouring countries including Qatar, Iran and Ethiopia; however, none of those mutations were associated with artemisinin resistance [36–38].

With regards to the partner drug, SP, the current study found a very high prevalence of the *pfdhfr* S108N and N51I mutations (84.8% each), with the *pfdhfr* double mutant (N51**I** + S108**N**) reported in 47% of the isolates while 37.8% carried the triple mutant haplotype (N51**I** + C59**R** + S108**N**). In 2012, a previous study on 176 isolates from Jazan region reported 33% and 34% prevalence of *pfdhfr* S108**N** and N51**I** mutations, respectively, with the double mutant (N51**I** + S108**N**) reported in 33% [22]. Interestingly, Bin Dajem et al. [22] found no mutations at other *pfdhfr* codons (C59**R** and I164**L**) and the *pfdhfr* triple mutated haplotype was not observed, whereas the current study detected C59**R** mutation in 37.7% of the isolates. In Taif region, a recent study among only 13 *P. falciparum* isolates reported the presence of *pfdhfr* double (N51**I** + S108**N**) and triple (N51**I** + C59**R** + S108**N**) mutations in three (23%) and nine (69%) isolates, respectively [23]. Moreover, results similar to those obtained by the current study have also been reported among imported *P. falciparum* isolates in the neighbouring country of Qatar [37]. By contrast, previous studies from Yemen have concluded that *P. falciparum* with the *pfdhfr* triple mutation (N51**I** + C59**R** + S108**N**) is not circulating in the country; however, none of these studies were conducted in areas bordering Jazan, Saudi Arabia [27, 39, 40].

In respect of *pfdhps*, the current study found a 56.3% and 51.7%% prevalence of mutations in the *pfdhps* gene at codons A437**G** and K540**E**, respectively, with 51.7% carrying the *pfdhps* double mutant haplotype (S**GE**AAI). Until 2016, a prevalence of K540**E** exceeding 50% had been reported in 12 African countries including Sudan, Somalia, Ethiopia, Tanzania and Kenya [11]. While Bin Dajem et al*.* [22] found a single mutation at the *pfdhps* codon A437**G** in only one isolate from Jazan in 2012, Soliman et al*.* [23] detected mutations at codon A437**G** in a single isolate (7.7%) and at codon K540**E** in five isolates (38.5%) from Taif region. While not found in the current study, mutations in the *pfdhps* gene at codons A581**G** and S436**A** have been detected in a single (7.7%) and seven (53.8%) isolates from the Taif region [23]. In Yemen, the results thus far have been mixed. Mutations in the *pfdhps* gene have not been detected by some studies [27, 39], whereas another previous study reported a prevalence of 44.7% of the *pfdhps* single mutation at codon A437**G** [40]. Interestingly, a novel mutation at codon I431**V** that may favour resistance risk has been found in west and sub-Saharan Africa as well as in imported isolates in the United Kingdom, Qatar and China [37, 41, 42]. However, this mutation (I431**V**) was not detected in the isolates investigated in the current study.

Bearing in mind that Bin Dajem et al*.* [22] conducted their study on 176 *P. falciparum* isolates collected from healthcare facilities in Jazan (i.e. a similar study area, setting and sample size to the current study), the present findings clearly imply an alarmingly increased prevalence (as well as emergence) in *pfdhfr* and *pfdhps* point mutations in Jazan region, particularly among Saudis, 11 years after the change in the malaria treatment policy. This study found a high prevalence of *pfdhfr* double (47%) and triple (37.8%) mutated haplotypes (i.e. 84.8% when combined as double-to-triple mutations) as well as a high prevalence of the *pfdhps* double mutant haplotype (51.7%). Interestingly, 23.8% of the isolates harboured a combination of the *pfdhfr* triple mutant (N51**I** + C59**R** + S108**N**) and the *pfdhps* double mutant (A437**G** + K540**E**) haplotypes (i.e. quintuple mutant genotype), a combination that confers full resistance to SP, according to a recent classification suggested by Naidoo and Roper [43].

The present study is the first to report the presence of quadruple and quintuple mutated *pfdhfr*–*pfdhps* genotypes in Saudi Arabia and the Arabian Peninsula in general. The distribution of different *pfdhfr* and *pfdhps* point mutations and related haplotypes across the governorates of Jazan region can be considered comparable, despite the absence of quintuple mutant genotypes in a few governorates as this could be due to the variation in the number of isolates collected from each area (Fig. 1 and Additional file 1: Table S2).

Previous studies conducted in different endemic countries have identified a strong association between the *pfdhfr* double mutation (N51**I** and S108**N**) and resistance to pyrimethamine [44, 45]. In the same vein, other previous studies have demonstrated that the triple mutant *pfdhfr* (N51**I** + C59**R** + S108**N**) confers a significant component of in-vitro and in-vivo resistance to SP [46, 47]. When this *pfdhfr* triple mutation is combined with a *pfdhps* double mutation, specifically A437**G** and K540**E**, producing either quadruple or quintuple mutant genotypes, the risk of SP treatment failure can be over 75% [48–50].

This study also found significant associations between the prevalence of mutated haplotypes and age, gender and nationality. Interestingly, the harbouring of *P. falciparum* with *pfdhfr*–*pfdhps* quintuple mutations (AC**IRN**I–S**GE**AAI) was significantly higher in patients aged ≥ 30 years compared to those aged below 30 years. The number of *pfdhfr* and *pfdhps* mutations was found to positively correlate with participants’ age, with an age of 20 years or older was identified as a risk factor of harbouring isolates with a high number of mutations [51]. By contrast, previous studies have concluded that age does not influence the distribution and carriage of resistant *P. falciparum* parasites whatever the type of mutation; however, these studies included only children aged ≤ 15 years [48, 52]. While the explanation for the association with gender is not known, it should be borne in mind that the identified association could be attributed to the low number of female participants involved in this study.

Interestingly, in Jazan region, the C59**R** *pfdhfr*-mutant allele was detected only in two out of 19 isolates in 2007 [19] and was not detected in any of 176 isolates (80% Saudi) in 2012 [22]. However, the current study found that the frequency of C59**R** point mutation and the quintuple AC**IRN**I–S**GE**AAI haplotype was significantly associated with participants’ nationality, with the frequency of AC**IRN**I–S**GE**AAI haplotype was almost double in the Saudi than non-Saudi patients. Despite the intensive efforts to eliminate indigenous malaria in the Kingdom, 52 (34.4%) cases in this study were Saudi. Moreover, as the majority of malaria cases in this study were among foreigners (65 from Yemen and 27 from Southern Asia, mainly Pakistan and India), the possibility of introducing ACT resistance into Saudi Arabia should not be ignored, especially as it has been over 10 years since the policy change to ACT for uncomplicated falciparum malaria treatment.

There are some limitations that should be considered when interpreting the findings of the present study. First, the total number of the collected *P. falciparum* isolates was small. Second, the small number of blood samples collected from some governorates as well as the small number of females compared to male participants. Third, the molecular findings were not further correlated with treatment outcome among the study participants. However, this study still provides important findings on both the types and prevalence of *P. falciparum* mutations circulating in southwestern Saudi Arabia and these findings will enable the setting up of a database on molecular markers of anti-malarial drug resistance in Jazan region. Furthermore, these findings “sound the alarm” for the Jazan region, which together with Aseer region, is one of the last remaining foci of malaria transmission in the country and therefore calls for the close monitoring of the efficacy of ACT.

## Conclusions

The current study reveals a high prevalence of the *pfdhfr* triple mutation (N51**I** + C59**R** + S108**N**) and *pfdhps* double mutation (A437**G** + K540**E**) in *P. falciparum* isolates from Jazan region, southwestern Saudi Arabia. It also found evidence of the presence of the related quadruple and quintuple mutated *pfdhfr*–*pfdhps* genotypes, which is the first time that these genotypes have been reported in Saudi Arabia and in the Arabian Peninsula in general. On the other hand, the study found an absence of *pfkelch13* mutations in *P. falciparum* isolates from the Jazan region. *Pfdhfr* and *pfdhps* mutations undermine the efficacy of SP partner drug, threatening the main ACT-based policy for falciparum malaria treatment in Saudi Arabia. Therefore, the continuous molecular and in-vivo monitoring of ACT in Jazan region is recommended in order to track the emergence and spread of parasites with reduced susceptibility to AS + SP. The findings of the current study provide a clear comprehensive picture of the level of anti-malarial drug resistance in the region. Hence it is hoped that these findings will contribute to the development of new strategies for therapeutic intervention against residual malaria in Saudi Arabia in order to achieve malaria elimination status.

## Supplementary information

**Additional file 1: Table S1.** Frequency of *pfdhfr* and *pfdhps* single mutant alleles for *P. falciparum* isolates from Jazan, Saudi Arabia according to demographic factors and parasitaemia. **Table S2.** Frequency distribution of *pfdhfr*, *pfdhps*, and combined *pfdhfr*–*pfdhps* mutant haplotypes for *P. falciparum* isolates from Jazan, Saudi Arabia according to governorates.

## Data Availability

The data that support the findings of this study are available from the corresponding author upon reasonable request.

## Abbreviations

ACT: Artemisinin-based combination therapy
*Pfdhfr*: *P. falciparum* Dihydrofolate reductase
*Pfdhps*: *P. falciparum* Dihydropteroate synthetase
*Pfkelch13*: *P. falciparum* Propeller domain of the Kelch 13 protein
AS: Artesunate
SP: Sulfadoxine–pyrimethamine
