# Supplementary material for: Increased prevalence of pfdhfr and pfdhps mutations associated with sulfadoxine–pyrimethamine resistance in Plasmodium falciparum isolates from Jazan Region, Southwestern Saudi Arabia: important implications for malaria treatment policy
Source: Malar J. 2020 Dec 2;19:446. doi: 10.1186/s12936-020-03524-x (PMC7709338; doi:10.1186/s12936-020-03524-x)
Supplement: Supplementary file 1 — Additional file 1: Table S1. Frequency of pfdhfr and pfdhps single mutant alleles for P. falciparum isolates from Jazan, Saudi Arabia according to demographic factors and parasitaemia. Table S2. Frequency distribution of pfdhfr, pfdhps, and combined pfdhfr–pfdhps mutant haplotypes for P. falciparum isolates from Jazan, Saudi Arabia according to governorates. [file 12936_2020_3524_MOESM1_ESM.docx]

**Table S1 Frequency of *pfdhfr* and *pfdhps* single mutant alleles for *P. falciparum* isolates from Jazan, Saudi Arabia according to demographic factors and parasitaemia.**

| **Marker*** | **Total** | **Age group** | | | **Sex** | | | **Nationality** | | | **Parasitaemia** | | |
| --- | --- | --- | --- | --- | --- | --- | --- | --- | --- | --- | --- | --- | --- |
|  |  | **< 30** | **≥ 30** | ***P*** | **Females** | **Males** | ***P*** | **Saudi** | **Non-Saudi** | ***P*** | **Low** | **Moderate-to-high** | ***P*** |
| ***Pfdhfr*** |  |  |  |  |  |  |  |  |  |  |  |  |  |
| N51**I** | 128 (84.8) | 59 (78.7) | 69 (90.8) | 0.038 | 22 (95.7) | 106 (82.8) | 0.115 | 42 (80.8) | 86 (86.9) | 0.322 | 71 (87.7) | 57 (81.4) | 0.288 |
| C59**R** | 57 (37.7) | 20 (26.7) | 37 (48.7) | 0.005 | 9 (39.1) | 48 (37.5) | 0.882 | 26 (50.0) | 31 (31.3) | 0.024 | 30 (37.0) | 27 (38.6) | 0.846 |
| S108**N** | 128 (84.8) | 59 (78.7) | 69 (90.8) | 0.038 | 22 (95.7) | 106 (82.8) | 0.115 | 42 (80.8) | 86 (86.9) | 0.322 | 71 (87.7) | 57 (81.4) | 0.288 |
| ***Pfdhps*** |  |  |  |  |  |  |  |  |  |  |  |  |  |
| A437**G** | 85 (56.3) | 40 (53.3) | 45 (59.2) | 0.467 | 17 (73.9) | 68 (53.1) | 0.064 | 33 (63.5) | 52 (52.5) | 0.198 | 44 (54.3) | 41 (58.6) | 0.601 |
| K540**E** | 78 (51.7) | 37 (49.3) | 41 (53.9) | 0.571 | 17 (73.9) | 61 (47.7) | 0.020 | 28 (53.8) | 50 (50.5) | 0.696 | 41 (50.6) | 37 (52.9) | 0.784 |

All values are number (%)

Parasitaemia levels: low (< 1,000 parasites/μl of blood); moderate-to-high (≥1,000 parasites/μl of blood)

* Mutant alleles are bold and underlined

Significant association (*P* < 0.05, Chi Square test)

**Table S2 Frequency distribution of *pfdhfr*, *pfdhps*, and combined *pfdhfr*–*pfdhps* mutated haplotypes for *P. falciparum* isolates from Jazan, Saudi Arabia according to governorates.**

| **Haplotypes** |  | **Baish** | **Abu Arish** | **Alharth** | **AlDair** | **Samtah** | **Sabya** | **Jizan** | **AlDarb** | **AlAridah** | **AlEidabi** | **Dhamad** | **Ahad Almsariha** |
| --- | --- | --- | --- | --- | --- | --- | --- | --- | --- | --- | --- | --- | --- |
| N |  | 32 | 18 | 18 | 15 | 15 | 12 | 10 | 9 | 9 | 5 | 4 | 4 |
| ***Pfdhfr*** |  |  |  |  |  |  |  |  |  |  |  |  |  |
| ACNCSI | Wild | 9.4 | 16.7 | 11.1 | 6.7 | 53.3 | 25.0 | 10.0 | 11.1 | 0.0 | 0.0 | 25.0 | 0.0 |
| AC**I**C**N**I | Double | 50.0 | 61.1 | 55.6 | 33.3 | 46.7 | 33.3 | 30.0 | 33.3 | 11.1 | 100.0 | 75.0 | 75.0 |
| AC**IRN**I | Triple | 40.6 | 22.2 | 33.3 | 60.0 | 0.0 | 41.7 | 60.0 | 55.6 | 88.9 | 0.0 | 0.0 | 25.0 |
| ***Pfdhps*** |  |  |  |  |  |  |  |  |  |  |  |  |  |
| SAKAAI | Wild | 43.8 | 16.7 | 55.6 | 53.3 | 46.7 | 33.3 | 40.0 | 66.7 | 22.2 | 40.0 | 100.0 | 50.0 |
| S**G**KAAI | Single | 3.1 | 5.6 | 11.1 | 0.0 | 6.7 | 0.0 | 10.0 | 0.0 | 11.1 | 0.0 | 0.0 | 0.0 |
| S**GE**AAI | Double | 53.1 | 77.7 | 33.3 | 46.7 | 46.7 | 66.7 | 50.0 | 33.3 | 66.7 | 60.0 | 0.0 | 50.0 |
| ***Pfdhfr*–*pfdhps*** |  |  |  |  |  |  |  |  |  |  |  |  |  |
| Combined haplotypes | Wild | 9.4 | 5.6 | 5.6 | 6.7 | 26.7 | 8.3 | 10.0 | 11.1 | 0.0 | 0.0 | 25.0 | 0.0 |
|  | Single | 0.0 | 0.0 | 0.0 | 0.0 | 6.7 | 0.0 | 0.0 | 0.0 | 0.0 | 0.0 | 0.0 | 0.0 |
|  | Double | 18.8 | 22.2 | 44.4 | 26.7 | 40.0 | 41.7 | 10.0 | 22.2 | 0.0 | 40.0 | 75.0 | 50.0 |
|  | Triple | 18.8 | 5.6 | 11.1 | 20.0 | 0.0 | 0.0 | 20.0 | 33.3 | 22.2 | 0.0 | 0.0 | 0.0 |
|  | Quadruple | 28.1 | 44.4 | 27.8 | 6.7 | 26.7 | 8.3 | 30.0 | 11.1 | 22.2 | 60.0 | 0.0 | 25.0 |
|  | Quintuple | 25.0 | 22.2 | 11.1 | 40.0 | 0.0 | 41.7 | 30.0 | 22.2 | 55.6 | 0.0 | 0.0 | 25.0 |

All values are %

N, number of isolates from each governorate

* Mutant alleles are bold and underlined
